# Enhancing Yield and Improving Grain Quality in Japonica Rice: Targeted EHD1 Editing via CRISPR-Cas9 in Low-Latitude Adaptation

**DOI:** 10.3390/cimb46040233

**Published:** 2024-04-22

**Authors:** Jian Song, Liqun Tang, Honghuan Fan, Xiaozheng Xu, Xinlu Peng, Yongtao Cui, Jianjun Wang

**Affiliations:** 1 Institute of Crops and Nuclear Technology Utilization, Zhejiang Academy of Agricultural Sciences, Hangzhou 310021, China; song521125@163.com (J.S.); liquntang2013@126.com (L.T.); xixi615@163.com (H.F.); cuiyongtao20@163.com (Y.C.); 2 College of Advanced Agriculture Sciences, Zhejiang A&F University, Hangzhou 311300, China; 19550178539@163.com (X.X.); 15057155749@163.com (X.P.)

**Keywords:** northeastern *Japonica*, heading date, CRISPR/Cas9, *Ehd1*, low latitude

## Abstract

The “*Indica* to *Japonica*” initiative in China focuses on adapting *Japonica* rice varieties from the northeast to the unique photoperiod and temperature conditions of lower latitudes. While breeders can select varieties for their adaptability, the sensitivity to light and temperature often complicates and prolongs the process. Addressing the challenge of cultivating high-yield, superior-quality *Japonica* rice over expanded latitudinal ranges swiftly, in the face of these sensitivities, is critical. Our approach harnesses the CRISPR-Cas9 technology to edit the *EHD1* gene in the premium northeastern *Japonica* cultivars Jiyuanxiang 1 and Yinongxiang 12, which are distinguished by their exceptional grain quality—increased head rice rates, gel consistency, and reduced chalkiness and amylose content. Field trials showed that these new *ehd1* mutants not only surpass the wild types in yield when grown at low latitudes but also retain the desirable traits of their progenitors. Additionally, we found that disabling *Ehd1* boosts the activity of *Hd3a* and *RFT1*, postponing flowering by approximately one month in the *ehd1* mutants. This research presents a viable strategy for the accelerated breeding of elite northeastern *Japonica* rice by integrating genomic insights with gene-editing techniques suitable for low-latitude cultivation.

## 1. Introduction 

The cultivation of high-yield crop varieties has significantly enhanced agricultural performance, particularly in adapting to diverse latitudes [1]. The photoperiod is a critical factor in this adaptation, as it significantly influences crop yields. In China, *Oryza sativa* L., predominantly a short-day (SD) crop, has evolved into two main subspecies: *indica* and *japonica* [2]. Traditionally, *indica* is cultivated in southern China’s lower-latitude areas, while *Japonica* is grown in the mid-latitude regions of the northeastern plain and along the Yangtze River [3]. Northeastern *japonica* rice is increasingly preferred for its superior grain quality. However, attempts to breed northeastern *Japonica*-like rice at lower latitudes have faced challenges due to photosensitivity or temperature sensitivity [4,5,6]. Selecting the optimal flowering time for southern China’s lower-latitude areas could enable the expansion of *Japonica* rice from north to south. However, traditional breeding methods are time-consuming [7]. Recent molecular genetic research has uncovered beneficial photoperiod-associated alleles in elite cultivars [8,9], paving the way for advanced gene-editing techniques, such as the clustered regularly interspaced short palindromic repeats (CRISPR)/CRISPR-associated 9 (Cas9) system [10,11].

Photoperiod conditions significantly promote rice heading [8]. Rice has evolved two distinct flowering pathways: a long-day (LD) flowering suppression pathway and a flowering-promotion pathway [8,12]. Under both LD and SD conditions, *Ehd1*, a B-type response regulator, plays a crucial role in integrating these signals. *Ehd1* enhances heading by elevating the expression of *Hd3a* and *RFT1* under SD conditions [13,14]. Various genes modulate the expression of *Ehd1*. For instance, *RID1*/*Ehd2*/*OsID1*, *SE5*, *OsMADS51*, and others positively influence *Ehd1* [15,16], while *Heading Date 1* (*Hd1*), *Ghd7*, *COL4*, and *DTH8*/*Ghd8*/*Hd5* have a negative impact [8,17,18,19,20]. *Hd1* is unique, suppressing heading under LD and promoting it under SD [17]. The roles of other key regulatory genes, like *Ghd7*, *Hd2*, and *DTH8*, are necessary for *Hd1* to reduce heading under LD [9,21]. *SE5* is involved in phytochrome chromophore biosynthesis [16]. Mutations in *se5* result in early heading under both SD and LD conditions. The *AP2* genes, containing the *miR172* target site, act as repressors of *Ehd1* [22]. Loss of *Ehd1* function leads to extended flowering times and exhibits elite agronomic traits at low latitudes, as demonstrated in the Longdao16 and Longdao24 rice varieties [3]. Therefore, *Ehd1* presents a potential target for genome editing to facilitate the adaptation of *Japonica* rice from north to south.

Addressing the pressing need to rapidly breed a diverse array of modern varieties, our study introduces a method using the CRISPR-Cas9 technique. We applied this technique to edit the *EHD1* gene in elite northeastern *Japonica* varieties, including Jiyuanxiang 1 and Yinongxiang 12, which are known for their superior grain quality (higher head rice rates; gel consistency; and lower chalky rice rates, chalkiness degree, and amylose content). The objectives include achieving suitable flowering times and elite plant architecture for modern rice production at low latitudes while preserving the superior grain quality traits of the progenitors. This endeavor represents an integration of gene editing with existing *Japonica* rice landraces and breeding strategies in northeastern China, aiming to enhance the adaptation of *Japonica* rice and breed new, modern varieties suitable for low-latitude regions.

## 2. Materials and Methods

### 2.1. Plant Materials and Growth Conditions

The transgenic plants used in this study were of the Yinongxiang and Jiyuanxiang varieties. All rice plants were cultivated in paddy fields under natural conditions at locations in Hangzhou and LingShui, managed by the Zhejiang Academy of Agricultural Sciences, China. 

### 2.2. Agronomic Trait Measurements

Upon reaching maturity, primary agronomic traits such as plant height, tiller count, leaf length, leaf breadth, spikelet length, number of primary and secondary branches, and seed-setting rate were evaluated. For each trait, data were collected from three biological replicates.

### 2.3. Plasmid Construction

The CRISPR/Cas9 system was utilized to target the *EHD1* gene, with two specific target sites designed. gRNA1EHD1 and gRNA2EHD1 were initially digested and then assembled into an intermediate vector [23]. This intermediate vector was subsequently incorporated into the pC1300-Cas9 binary vector. Details of the target sequences can be found in Supplementary Table S3. The binary vector loaded with two sgRNAs was employed for genetic transformation using the Agrobacterium-mediated transformation method (strain EHA105), as outlined in previous studies [24]. 

### 2.4. Detection of Mutations

Genomic DNA was extracted from approximately 100 mg of leaf tissue from the transgenic rice plants by using the CTAB method. PCR amplification of the fragments surrounding the target sites was performed by using KOD FX DNA polymerase (Toyobo Co., Ltd., Osaka, Japan). The amplified DNA fragments were then sequenced using the Sanger method (Applied Biosystems, Foster City, CA, USA) and analyzed by the degenerate sequence decoding method [25,26]. To identify transgene-free plants, PCR amplification was conducted by using specific primers for the hygromycin phosphotransferase gene.

### 2.5. RNA Extraction and RT-PCR

Total RNA was extracted from leaf tissues by using TRIzol reagent (Thermo Fisher Scientific, Waltham, MA, USA). First-strand cDNA synthesis was performed by using 1 μg of RNA and the ReverTra Ace qPCR RT Master Mix with gDNA Remover (Toyobo Co., Ltd., Osaka, Japan). Real-time PCR was conducted on each cDNA sample in duplicate. The quantitative real-time PCR experiments were performed by using the Power SYBR Green PCR Master Mix kit (Applied Biosystems, Foster City, CA, USA) under specified conditions. The rice UBIQUITIN gene served as an internal control, and relative expression ratios were calculated by using the 2^−ΔΔCT^ method. Gene-specific primers are listed in Table S3.

### 2.6. Photoperiod and Temperature Treatments

*ycas-1*, *jcas-2*, and WT plants were sown on 15 June 2022 at the Institute of Crops and Nuclear Technology Utilization, Zhejiang Academy of Agricultural Sciences. Fifty-eight-day-old plants underwent photoperiod and temperature treatments in growth chambers. Plants were treated for 7 days and sampled randomly every 3 days over a 10-day period, with each sampling involving three biological replicates.

### 2.7. Measurement of Amylose, Gel Consistency, and Alkali Spreading Value

Amylose content and gel consistency in mature grains of WT, *ycas-1*, and *jcas-2* plants were measured by using the concanavalin A-based method (Sigma-Aldrich, St. Louis, MO, USA). Gel consistency was determined by the alkali digestion test (Sigma-Aldrich, St. Louis, MO, USA), which involved evaluating starchy endosperm by using a standard seven-point numerical spreading scale. The alkali spreading value (ASV) classified the gelatinization temperature (GT) of rice grains into four groups: high (1–2), high–intermediate (3), intermediate (4–5), and low (6–7)

### 2.8. Statistical Analysis

Data were expressed as mean values ± standard deviations (SDs) based on three biological replicates. Statistical significance was determined by using Student’s unpaired *t*-test and Duncan’s multiple range test. Significance levels were set at 5% (indicated by a single asterisk, *) and 1% (indicated by a double asterisk, **) for the *t*-test. For Duncan’s multiple range test, significance levels were also set at 5% and 1%, and these are indicated in the respective figures and tables with different letters to denote statistically significant differences among the groups.

## 3. Results

### 3.1. Early Flowering of Northeastern Japonica Rice at Low Latitudes

A study involving 119 high-grain-quality northeastern *Japonica* rice varieties in Hangzhou, a low-latitude region, revealed that these varieties flower 30–50 days earlier than the local cultivars, Zhe 08A and Zhejing 99 (Figure S1, Table S1). This early flowering, however, limits their direct application in low-latitude regions. The findings suggest the need for improvement in genes associated with the long-day flowering suppression pathway.

### 3.2. Both ehd1-Ji Yuan Xiang 1 and ehd1-Yi Nong Xiang 12 Display Delayed Heading Date and Good Field Performance

**Figure 1:**
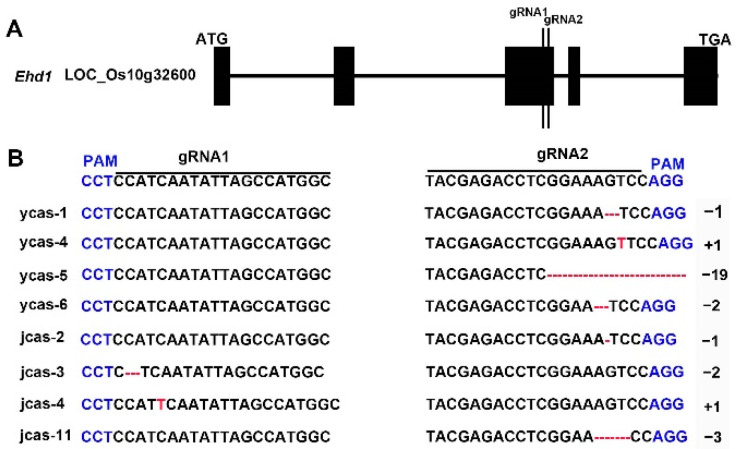
Illustration of *ehd1* mutant alleles in Jiyuanxiang 1 and Yinongxiang 12 varieties. (**A**) Targeted *EHD1* locus for CRISPR-Cas9 editing; (**B**) alignment of mutational sequences in the newly created *ehd1* alleles within the backgrounds of Jiyuanxiang 1 and Yinongxiang 12, compared with the wild type (WT) sequence. Protospacer Adjacent Motifs (PAMs) are marked in blue for clarity.

Among the varieties studied, Ji Yuan Xiang 1 and Yi Nong Xiang 12 were selected for modification of their flowering time. We employed the CRISPR/Cas9 system to target the *EHD1* gene in these varieties, building upon previous research for the construction of the CRISPR/Cas9 vector. The gRNA1 and gRNA2 targeting sites were strategically designed within the third exon of the *EHD1* gene (Figure 1A). Initial steps involved cloning the target sequences into SK-gRNA vectors, followed by assembling these gRNAs into a single intermediate vector to create a combined SK–gRNA1EHD1–gRNA2EHD1 structure. Subsequently, the sgRNAs were incorporated into the pC1300-Cas9 expression vector for genetic transformation. This resulted in 21 and 38 positive transgenic plants for Yinongxiang 12 and Jiyuanxiang 1, respectively, in the T0 generation (Table S2). The sequencing of the target regions in these plants revealed mutation rates of 0% and 47.6% at the gRNA1 and gRNA2 targeting sites in Yinongxiang 12, and 7.9% and 34.2% in Jiyuanxiang 1, respectively. These results demonstrate that the CRISPR/Cas9-mediated editing efficiency significantly varies between the two rice varieties Jiyuanxiang 1 and Yinongxiang 12, highlighting the influence of distinct genetic backgrounds on gene-editing outcomes. This underscores the necessity for tailored gene-editing strategies when targeting specific traits within different agricultural varieties.

To assess the phenotypic consequences of the new *ehd1* mutations and eliminate selective marker genes and T-DNA elements, T0 progeny plants underwent DNA sequencing and were self-pollinated to produce T1 progeny. T2 progeny, either homozygous for *ehd1*/*ehd1* or free of T-DNA, were subjected to phenotypic screening and genotyping (Figure S2). The sequencing analysis identified deletions or insertions at the predicted editing sites in *ycas-1*, *ycas-4*, *ycas-5*, *ycas-6*, *jcas-2*, *jcas*-3, *jcas*-4, and *jcas*-11, potentially leading to altered protein functions (Figure 1B). 

**Figure 2:**
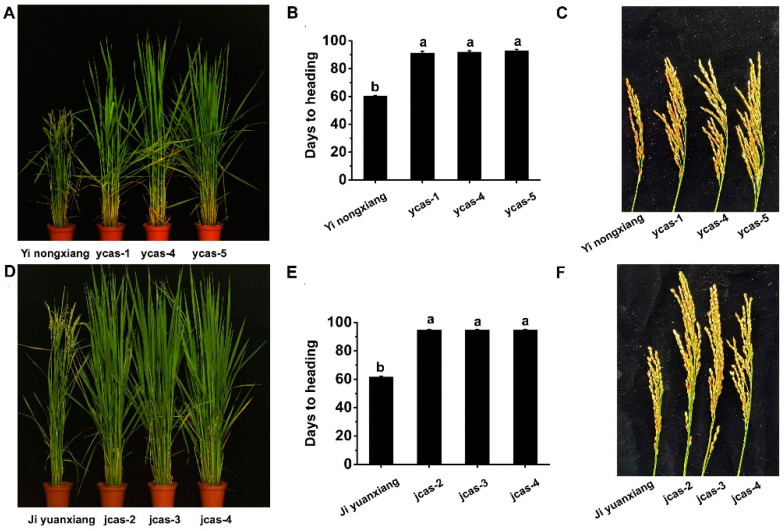
Characteristics of *ehd1* mutants derived from CRISPR-Cas9 editing with Jiyuanxiang 1 and Yinongxiang 12. (**A**) Physical characteristics of various *ehd1* mutants in the Yinongxiang 12 background, developed through gene editing. Scale bar represents 20 cm. (**B**) Extended basic vegetative growth periods observed in *ycas9-1*, *ycas9-4*, and *ycas9-5*. (**C**) Comparative analysis of panicle length between the wild type (Jiyuanxiang 1) and *jcas9-2*, *jcas9-3*, and *jcas9-4*. (**D**) Physical characteristics of different *ehd1* mutants with the Jiyuanxiang 1 background, developed through gene editing. Scale bar represents 20 cm. (**E**) Prolonged basic vegetative growth periods seen in *jcas9-2*, *jcas9-3*, and *jcas9-4*. (**F**) Panicle length comparison between the wild type (Jiyuanxiang 1) and *jcas9-2*, *jcas9-3*, and *jcas9-4*. Error bars represent means ± SDs (*n* = 3). Statistical analysis of agronomic trait variations was performed by using Duncan’s multiple range test (*p* < 0.05). The letters ‘a’ and ‘b’ indicate a highly significant difference.

To investigate the impact of these allelic variations on yield, several agronomic traits were measured (Table 1). For instance, the average plant height of the mutants ranged from 102.7 cm to 111.7 cm (Figure 2A,D), showing a noticeable increase compared with the WT. Furthermore, the grain number per panicle in all new *ehd1* alleles demonstrated varying degrees of increase relative to the WT (Figure 2C,F). The number of tillers per plant in the mutant lines also saw a significant increase compared with the WT. Notably, the heading date in *ehd1* mutants was delayed by about 30 days (Figure 2B,E). In yield tests, the average yield of *ycas-1* and *jcas-2* across thirty plants each surpassed that of the WT (Yinongxiang 12 and Jiyuan xiang 1) (Table 1). The next phase involves applying these new *ehd1* alleles in breeding and fertilizer testing to maximize yield potential.

### 3.3. Temperature Sensitivity of Modified ehd1 Variants

**Figure 3:**
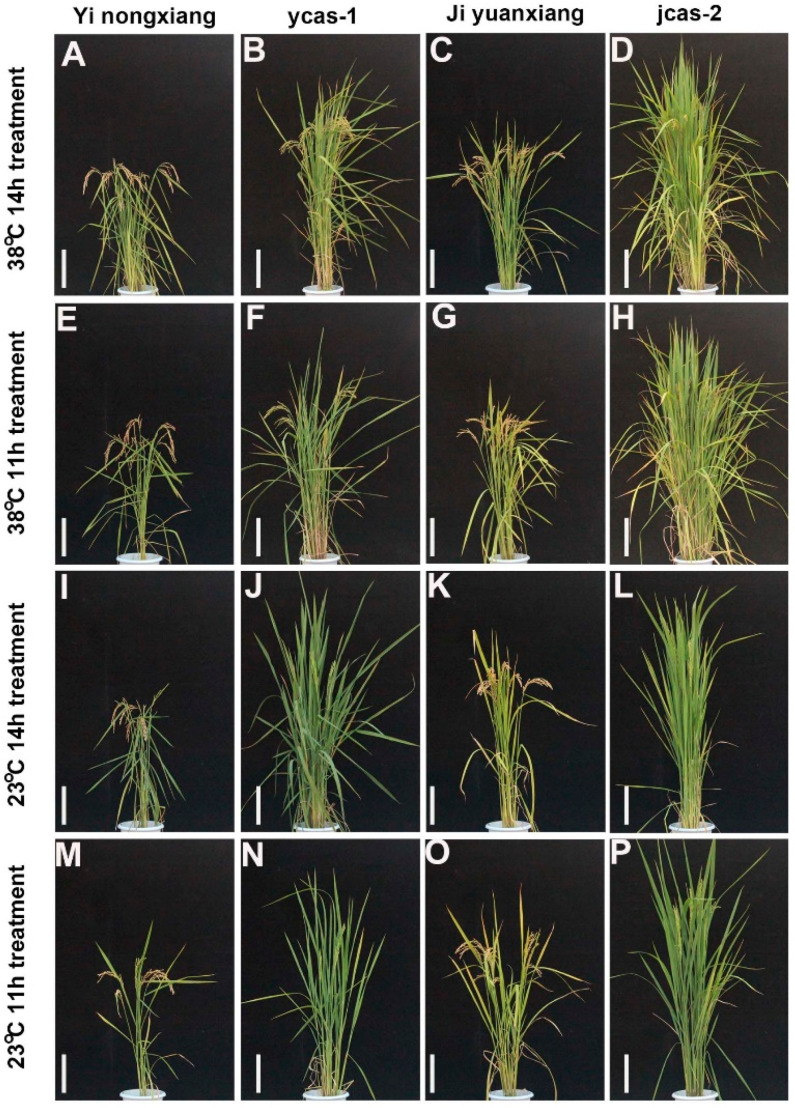
Temperature sensitivity in *ehd1* mutants of Jiyuanxiang 1 and Yinongxiang 12. (**A**) Trait of Yi nongxiang under 38 °C, 14 h treatment. (**B**) Trait of ycas-1 under 38 °C, 14 h treatment. (**C**) Trait of Ji yuanxiang under 38 °C, 14 h treatment. (**D**) Trait of *jcas-2* under 38 °C, 14 h treatment. (**E**) Trait of Yi nongxiang under 38 °C, 11 h treatment. (**F**) Trait of *ycas-1* under 38 °C, 11 h treatment. (**G**) Trait of Ji yuanxiang under 38 °C, 11 h treatment. (**H**) Trait of *jcas-2* under 38 °C, 11 h treatment. (**I**) Trait of Yi nongxiang under 23 °C, 14 h treatment. (**J**) Trait of *ycas-1* under 23 °C, 14 h treatment. (**K**) Trait of Ji yuanxiang under 23 °C, 14 h treatment. (**L**) Trait of *jcas-2* under 23 °C, 14 h treatment. (**M**) Trait of Yi nongxiang under 23 °C, 11 h treatment. (**N**) Trait of *ycas-1* under 23 °C, 11 h treatment. (**O**) Trait of Ji yuanxiang under 23 °C, 11 h treatment. (**P**) Trait of *jcas-2* under 23 °C, 11 h treatment. Bar = 20 cm.

**Figure 4:**
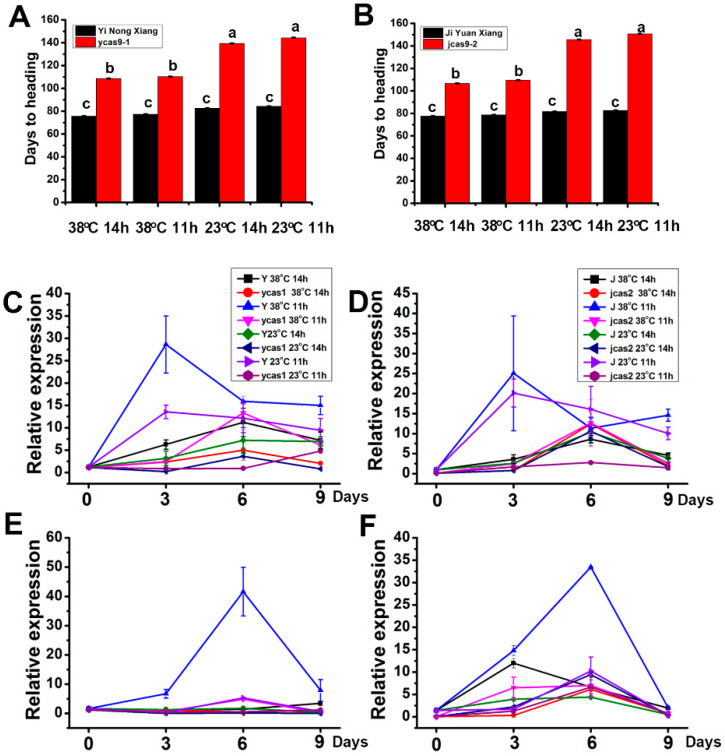
Heading dates and expression patterns of *Hd3a* and *RFT1* in the WT. Analysis of WT and mutant lines under different temperature and photoperiod conditions: artificial high temperature and long day (AHTLD), artificial high temperature and short day (AHTSD), artificial low temperature and long day (ALTLD), and artificial low temperature and short day (ALTSD). The study involved fifty-eight-day-old plants. (**A**,**B**) Heading day comparisons between Jiyuanxiang and *jcas9-2*, and Yinongxiang and *ycas9-1*, under varied light and temperature treatments (AHTLD, AHTSD, ALTLD, and ALTSD); (**C**–**F**) Expression patterns of *Hd3a* and *RFT1* in the presence and absence of *Ehd1* (Jiyuanxiang vs. *jcas9-2*; Yinongxiang vs. *ycas9-1*). Error bars represent means ± SDs (n = 3). Statistical analysis of agronomic trait variations was performed by using Duncan’s multiple range test (*p* < 0.05). The letters ‘a’, ‘b’, and ‘c’ indicate a highly significant difference.

To investigate the effects of *ehd1* mutations on photoperiod and temperature responses, experiments with *ycas-1* and *jcas-2* mutants were conducted. The results show that variations in photoperiods and temperatures impacted the mutants differently from the WT (Figure 3 and Figure 4A,B). Both *ycas-1* and *jcas-2*, along with the WT, demonstrated temperature sensitivity; notably, *ycas-1* and *jcas-2* exhibited extended basic vegetative growth periods at 23 °C compared with 38 °C. Similarly, under photoperiod treatments, these mutants showed marginally longer basic vegetative growth periods under 11 h light conditions than under 14 h light conditions (Figure 3 and Figure 4A,B).

Additionally, the mRNA expression levels of *Hd3a* and *RFT1* were monitored in *ycas-1* and *jcas-2* under various photoperiod and temperature conditions. Initially, the expression levels of these genes in the mutants were akin to those in the WT. However, a significant increase was observed after 3 to 6 days of treatment (Figure 4C–F). This pattern suggests a negative correlation between the heading date and the expression levels of *Hd3a* and *RFT1*. This finding supports the hypothesis that the transcriptional activation of these genes largely depends on the presence of functional *Ehd1*. The observed photoperiod and temperature responses indicate that a loss of *Ehd1* function leads to the inactivation of *Hd3a* and *RFT1* expression, thereby resulting in a phenotype characterized by delayed flowering.

### 3.4. High Grain Yield and Quality of ehd1 Mutants in Low-Latitude Regions

The superior grain quality of *japonica* rice has significantly boosted its popularity in China. However, extended periods of basic vegetative growth can negatively impact both grain yield and quality. This study evaluated the grain quality of the ycas-1 and *jcas-2* variants by analyzing several grain quality characteristics, such as chalkiness degree, chalky rice rate, amylose content, gel consistency, and alkali spreading value. These characteristics were compared across Ji Yuan Xiang 1, Yi Nong Xiang 12, ycas-1, and *jcas-2* (Table 2). The results indicate that ycas-1 and *jcas-2*, cultivated in Hangzhou, showed a lower chalky rice rate and chalkiness degree than Ji Yuan Xiang 1 and Yi Nong Xiang 12, with no significant differences in other traits (*t*-test, *p* > 0.1). The reduced chalky rice rate and chalkiness degree in the *ehd1* mutant lines, *ycas-1* and *jcas-2,* might be attributed to the lower temperatures experienced during their grain-filling stage. Notably, the *ycas-1* and *jcas-2* lines demonstrated commendable grain quality when cultivated in Hangzhou.

## 4. Discussion

### 4.1. Advancements in Genome Editing for Rice Breeding

This study highlights the pivotal role of genome editing in rice gene research, breeding, and domestication. The CRISPR/Cas9 system, consisting of Cas9 protein and a single-stranded guide RNA (sgRNA), is now foundational in this field [11,27]. This technique, including methods like Prime genome editing and CRISPR/Cas9-based base editing, has seen successful application across diverse crops, like rice, sorghum, wheat, tomato, and maize [11,28,29,30,31,32,33]. Rice, with its small genome size, high transformation efficiency, extensive quality reference sequences, and varied genomic haplotypes, serves as an ideal model for genetic studies and breeding [8]. Numerous genes have been targeted for improvement in rice by using CRISPR/Cas9, including those associated with disease resistance, grain quality, and yield [11,31,34].

### 4.2. Tailoring Northeastern Japonica Rice for Low-Latitude Cultivation

Photoperiodic gene adaptation in rice is crucial for its suitability across different latitudes and directly impacts grain yield [9]. Single-nucleotide polymorphisms (SNPs) in these genes vary among varieties at different latitudes [35]. Studies have focused on latitude adaptation in rice through enhanced expression or mutations in flowering time genes [27]. For instance, the allele of *Heading date 1* (*Hd1*) in *indica* varieties has been backcrossed into a *Japonica* variety, facilitating the integration of *indica* traits into *Japonica* rice in southern China. Mutations in the *Ehd1* gene, particularly in its third exon, have shown promising results in delaying the heading date and enhancing suitability for low-latitude growth [3,27].

The adaptation of photoperiodic genes is crucial for the suitability of rice cultivation across different latitudes, which directly impacts grain yield. In our study, we focused on the adaptation of rice to low latitudes through modifications in the Ehd1 gene, which is instrumental in delaying flowering time. These genetic modifications are particularly important for northeastern *Japonica* rice varieties, which, despite their superior grain quality, face challenges in breeding due to their sensitivity to light and temperature.

The transformation of Jiyuanxiang 1 and Yinongxiang 12, varieties known for their high-quality grain, through CRISPR-Cas9-mediated editing demonstrates the potential to overcome these environmental sensitivities. By introducing mutations in the *Ehd1* gene, we delayed the flowering period to a time that avoids the peak temperatures of August, which typically range from an average low of 27 °C to a high of 35 °C. Instead, the edited varieties now flower in late August, with grain filling occurring in September, under milder temperature conditions of 22 °C to 29 °C. This strategic delay in the flowering time circumvents the high-temperature stress during the crucial grain-filling period, markedly improving grain quality. This evidence showcases the power of genome editing in mitigating adverse temperature effects on crop yield and quality and underscores the importance of precise genetic modifications for enhancing crop resilience and productivity in response to climate variability.

Furthermore, the observed differences in gene-editing efficiency between gRNA1 and gRNA2 could be attributed to variations in target site accessibility, sgRNA design, or genomic context, which may influence the CRISPR/Cas9 system’s ability to induce precise edits. Understanding these nuances can guide improvements in sgRNA design and editing strategies, enhancing the precision and efficiency of genome editing applications in rice and other crops.

In summary, the modification of the *EHD1* gene in northeastern *japonica* rice varieties has proven effective for producing high-quality *Japonica* rice adapted to low latitudes. With the flowering times of the *ycas-1* and *jcas-2* variants closely aligning with local main varieties in Hangzhou, future efforts will focus on the broader application of these genetically edited varieties in rice breeding programs for low-latitude regions, thereby meeting the demands for high-quality rice production. This approach could serve as a blueprint for similar advancements in other crop varieties, highlighting the transformative impact of precision breeding technologies in modern agriculture.

## Figures and Tables

**Table 1:** Major agronomic traits of the *ehd1* mutant lines.

Rice Line	PH (cm)	PNPP	PL (cm)	PB	SB	SPP	SSR (%)
Yinongxiang 12	78.3 ± 2.1 a	7.3 ± 0.5 a	16.7 ± 0.3 a	5 ± 1.7 a	20.7 ± 1.5 a	76.3 ± 1.5 a	88.6 ± 1.9
*ycas-1*	102.7 ± 1.5 b	17.3 ± 0.6 b	23.2 ± 1.0 b	11.3 ± 0.6 b	40.3 ± 2.1 b	146.3 ± 10.2 b	88.3 ± 6.7
*ycas-4*	102.3 ± 1.2 b	19.3 ± 1.5 b	23.8 ± 0.3 b	12 ± 1 b	42.6 ± 4.0 b	148.7 ± 9.8 b	91.6 ± 3.2
*ycas-5*	103.7 ± 1.5 b	16.3 ± 1.5 b	23.7 ± 0.3 b	12 ± 1.7 b	44.3 ± 3.1 b	145.3 ± 6.0 b	87.5 ± 2.1
Jiyuanxiang 1	83.3 ± 1.2 a	10.3 ± 1.5 a	17.6 ± 0.3 a	10.0 ± 1.7 a	9.7 ± 1.2 a	99.3 ± 1.5 a	83.9 ± 3.5
*jcas-2*	109.3 ± 0.6 b	18.7 ± 3.1 b	27 ± 0.1 b	14.3 ± 1.5 b	25.3 ± 2.5 b	227.7 ± 14.3 b	92.0 ± 1.6
*jcas-3*	110.7 ± 1.5 b	16.7 ± 1.5 b	27.1 ± 0.3 b	14.0 ± 2.6 b	26.3 ± 1.2 b	229.3 ± 2.5 b	89.7 ± 1.7
*jcas-4*	111.7 ± 2.1 b	19.3 ± 1.5 b	26.6 ± 0.4 b	14.6 ± 2.1 b	22.6 ± 2.9 b	223.3 ± 5.9 b	91.2 ± 1.1

Note: The table presents average values for plant height (PH), panicle number per plant (PNPP), panicle length (PL), primary branches (PB), secondary branches (SB), spikelets per panicle (SPP), and seed-setting rate (SSR) with standard deviations (±SDs; *n* = 10). Statistical analysis of agronomic trait variations was performed by using Duncan’s multiple range test (*p* < 0.05). The letters ‘a’ and ‘b’ indicate a highly significant difference between the two.

**Table 2:** Comparative analysis of rice quality characteristics in *ehd1* mutant lines.

	Brown Rice Rate (%)	Milled Rice Rate (%)	Head Rice Rate (%)	Grain Length (mm)	Chalkiness Grain Rate (%)	Chalkiness (%)	Amylose (%)	Gel Consistency (mm)	Alkali Spreading Value
Yi nongxiang	81.41 ± 0.47	72.78 ± 0.72	69.85 ± 1.41	5.97 ± 0.05	52.67 ± 3.51 **	12.07 ± 1.66 **	17.46 ± 0.24 **	55.67 ± 5.13	6.60 ± 1.10
*ycas9-1*	82.99 ± 0.279	72.59 ± 0.29	67.51 ± 0.75	6.21 ± 0.01	44.00 ± 2.65	7.70 ± 0.25	15.51 ± 0.44	58.00 ± 3.60	6.50 ± 0.00
Ji yuanxiang	82.08 ± 0.44	74.38 ± 0.62	73.77 ± 0.78	4.63 ± 0.02	62.33 ± 7.64 **	20.03 ± 2.60 **	14.57 ± 0.31	62.67 ± 0.58	6.50 ± 0.00
*jcas9-2*	83.46 ± 0.31	74.40 ± 0.41	73.36 ± 0.43	4.67 ± 0.02	32.67 ± 1.53	3.70 ± 0.44	14.65 ± 0.16	62.67 ± 3.06	6.70 ± 0.17

Data represent the means ± SDs of three biological replicates (Student’s *t*-test: ** *p* < 0.01).

## Data Availability

The original data of this present study are available from the corresponding authors.

## Supplementary Materials

The following supporting information can be downloaded at: https://www.mdpi.com/article/10.3390/cimb46040233/s1, Figure S1: Frequency distribution of heading date of the elite northeastern plain japonica varieties and two control japonica varieties. Analysis performed using GraphPad Prism software (GraphPad Software, San Diego, CA, USA); Figure S2: Identification of hygromycin in different edited plants, Hygromycin detection performed using ELISA Kit (Sigma-Aldrich, St. Louis, MO, USA); Table S1: The heading date of elite northeastern plain japonica varieties in the Yangtze River region; Table S2: The number of plants detected in the two Yi Nong Xiang 12 PAM sites and Ji Yuan Xiang 1 PAM sites; Table S3: Primer sequences used in this study, Primers synthesized by Integrated DNA Technologies (IDT, Coralville, IA, USA).
