# Supplementary material for: Enhancing Yield and Improving Grain Quality in Japonica Rice: Targeted EHD1 Editing via CRISPR-Cas9 in Low-Latitude Adaptation
Source: Curr Issues Mol Biol. 2024 Apr 22;46(4):3741–51. doi: 10.3390/cimb46040233 (PMC11049033; doi:10.3390/cimb46040233)
Supplement: Supplementary file 1 [file cimb-46-00233-s001.zip › cimb-2956603-supplementary.pdf]

Table S1.

The heading date of elite northeast plain japonica varieties in Yangtze River region.

Variety	Sowing	Heading	Heading day(day)
Kong Yu 163	5/28	7/20	53
Long Jing 180	5/28	8/3	67
Long Jing 31	5/28	7/20	53
Long Qing Dao 3	5/28	7/20	53
Kong Yu 131	5/28	7/21	54
He Jiang 19	5/28	7/20	53
Tong 35	5/28	7/30	63
Long Jing 47	5/28	8/1	65
Dong Nong 418	5/28	7/21	54
Xiang Shui 1	5/28	7/30	63
Xiang Shui 2	5/28	8/4	68
Zhong Ke Fa 6	5/28	8/3	67
Xiang Shui 4	5/28	8/2	66
Zhong Ke 804	5/28	8/4	68
Xiang Shui 918	5/28	7/26	59
Zhong Ke Fa 5	5/28	8/6	70
Zhen Bao Dao 1	5/28	7/28	61
Long Jing 21	5/28	7/18	51
Long Dao 856	5/28	8/6	70
Ken Xiang Dao	5/28	7/24	57
Long Dao 3	5/28	7/20	53
Ken Dao 10	5/28	7/27	60
Long Dao 20	5/28	7/28	61
Lian Dao 1	5/28	8/3	67
Kong Yu 151	5/28	7/20	53
Lian Dao 2	5/28	7/22	55
Long Ken 257	5/28	7/23	56
Shui Dao 3	5/28	7/21	54
Long Jing 57	5/28	7/21	54
5 You Dao 4	5/28	8/4	68
Qing Dao 15-851	5/28	7/20	53
Mu Dan Jiang 30	5/28	7/19	52
Long Ken 263	5/28	7/19	52
San Jiang 12	5/28	8/5	69
San Jiang 6	5/28	7/20	53
Qing Dao 13-851	5/28	7/20	53
Nong Da 3811	5/28	7/20	53
Nong Da 3045	5/28	7/20	53
Qiu Guang	5/28	7/20	53
Miao Xiang Dao 1	5/28	7/20	53
Long Jing 26	5/28	7/19	52

Long Ken 222	5/28	7/20	53
Long Jing 31	5/28	7/20	53
Long Jing 25	5/28	7/29	62
Long Ken 262	5/28	7/20	53
Long Ken 261	5/28	7/20	53
Long Jing 43	5/28	7/20	53
Long Jing 29	5/28	7/20	53
Long Jing 27	5/28	7/27	60
Ken Yu 10427	5/28	7/21	54
Long Jing 217	5/28	7/20	53
Long Jing 21	5/28	7/20	53
Long Jing 20	5/28	7/20	53
Kong Yu 101	5/28	7/20	53
Long Jing 201	5/28	7/20	53
Long Dao 18	5/28	7/20	53
Ken Dao 179	5/28	7/21	54
Len Dao 1608	5/28	7/23	56
Ken Jing 161	5/28	7/22	55
Ken Dao 1	5/28	7/23	56
Ken Dao 12-805	5/28	7/21	54
Ken Dao 20122133	5/28	7/19	52
Qiangj Ing18	5/28	7/26	59
Long Jing 46	5/28	7/21	54
Ken Yan 02	5/28	8/1	65
Ken Yan 03	5/28	7/21	54
Ken Yan 04	5/28	7/20	53
Ken Yan 05	5/28	7/20	53
Ken Jing 5	5/28	7/19	52
Ken Dao 87	5/28	7/20	53
Ken Jing 1609	5/28	7/20	53
Ken Jing 12	5/28	7/31	64
Ken Dao 12489	5/28	7/21	54
Kan Dao 95	5/28	7/31	64
Kan Dao 92	5/28	7/20	53
Kan Dao 91	5/28	7/19	52
Kenjing 6	5/28	7/19	52
Ken Dao 61	5/28	7/29	62
Ken Dao 63	5/28	7/20	53
Ken Dao 70	5/28	7/20	53
Ken Dao 73	5/28	8/2	66
Ken Dao 81	5/28	7/20	53
Kan Dao 85	5/28	7/20	53
Kan Dao 86	5/28	7/19	52
Ken Dao 9	5/28	7/19	52

Kan Dao 90	5/28	7/18	51
Kan Dao 19	5/28	7/19	52
Kan Dao 20	5/28	7/20	53
Kan Dao 23	5/28	7/21	54
Kan Dao 25	5/28	7/22	55
Kan Dao 26	5/28	7/21	54
Kan Dao 20	5/28	7/20	53
Kan Dao 30	5/28	7/20	53
Kan Dao 41	5/28	7/21	54
Ken Dao 46	5/28	7/24	57
Kan Dao 60	5/28	7/21	54
Ken 11-2061	5/28	7/22	55
Ken 11-913	5/28	7/21	54
Ken 13-1628	5/28	8/4	68
Ken 13-1646	5/28	7/20	53
Ken 143202	5/28	7/21	54
Ken 14387	5/28	7/21	54
Ken Dao 11-299	5/28	7/22	55
Ken Dao 12	5/28	7/26	59
Kan Dao 1511	5/28	7/24	57
Kan Dao 17	5/28	8/1	65
Dong Nong 427	5/28	7/23	56
Fu 004	5/28	7/21	54
Fu He 2	5/28	7/21	54
Jian 08-603	5/28	7/21	54
Jian Jing 1202	5/28	7/18	51
Ken 10-1620	5/28	8/1	65
Wu You Dao 4	5/28	8/4	68
Song Jing 29	5/28	7/28	61
Suijing 109	5/28	8/6	70
Qijing 10	5/28	8/5	69
Longjing 1755	5/28	8/1	65
Ji Yuan Xiang 1	5/28	7/28	61
Yi Nong Xiang 12	5/28	7/27	60
Zhe 08B	5/28	9/6	101
Zhejing 99	5/28	8/28	92

Table S2.

The number of plants detected in the two Yi Nong Xiang 12 PAM sites and Ji Yuan Xiang 1 PAM sites.

Variety	No. of transgenic plants	No. of plants with gRNA1 mutations(%)*	No. of plants with gRNA2 mutations(%)*
Yi Nong Xiang 12	21	0	10(47.6)
Ji Yuan Xiang 1	38	3(78.9)	13(34.2)

Table S3.

Primer sequences used in this study.

Primer name	Primer sequence (5'-3')
RFT1-qRT F	CGTGATGGTAGACCCGGATG
RFT1-qRT R	GCCCAAATGTTGCTCCAGTG
Hd3a-qRT F	TAGGGTTGTGGGTGATGTGC
Hd3a-qRT R	CCATTGGACACGGTCTTGGA
OsUBQ-F	AACCAGCTGAGGCCCAAGA
OsUBQ-R	ACGATTGATTTAACCAGTCCATGA
Ehd1-H1-gRNA	CCTCCATCAATATTAGCCATGGC
Ehd1-H2-gRNA	TACGAGACCTCGGAAAGTCCAGG
Ehd1 -site-idF	GGATCGAAGAGCTGAGCAAC
Ehd1 -site-idR	GATCACTCACTGTCTTCTCCG

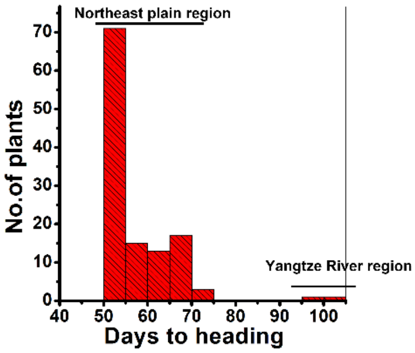

Figure S1. Frequency distribution of heading date of the elite northeast plain *japonica* varieties and two control *japonica* varieties.

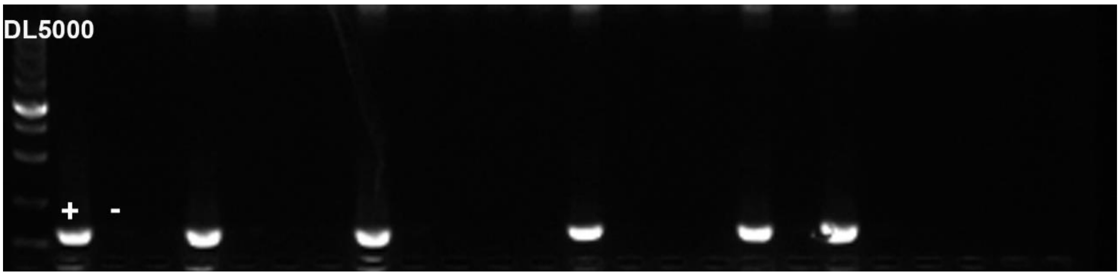

Figure S2: Identification of hygromycin in different edited plants.
